# Metaphorical expressions originating from human senses: Psycholinguistic and affective norms for German metaphors for internal state terms (MIST database)

**DOI:** 10.3758/s13428-021-01639-w

**Published:** 2021-07-08

**Authors:** Nadine Müller, Arne Nagels, Christina Kauschke

**Affiliations:** 1 Department of German Linguistics, University of Marburg, Pilgrimstein 16, 35032 Marburg, Germany; 2 Department of English and Linguistics, Johannes Gutenberg-University Mainz, Mainz, Germany

**Keywords:** Metaphor, Database, German, Internal states, Emotion, Source domains, Valence, Arousal, Familiarity, Sentence completion

## Abstract

Internal states, e.g., emotions, cognitive states, or desires, are often verbalized by figurative means, in particular by embodied metaphors involving human senses, such as touch, taste, and smell. The present paper presents a database for German metaphorical expressions conveying internal states with human senses as their source domains. 168 metaphorical expressions from the source domains of vision, hearing, smell, taste, touch, and temperature combined with literal equivalents were collected and rated by 643 adults. The agreement between the metaphor and an equivalent literal expression, as well as emotional valence, arousal, and familiarity values were assessed using a 7-point Likert scale. Between the metaphorical expressions and their equivalents, familiarity, but not valence or arousal differed significantly while agreement ratings indicated high similarity in meaning. The novel database offers carefully controlled stimuli that can be used in both empirical metaphor research and research on internal state language. Using part of the stimuli in a sentence completion experiment revealed a significant preference for literal over metaphorical expressions that cannot be attributed to higher familiarity levels.

## Introduction

Corpus analyses show that on average, each seventh lexical unit in academic texts, news, fiction, and conversation is related to metaphor (Steen, Dorst, Herrmann, Kaal, & Krennmayr, 2010). With the rise of conceptual metaphor theory (Kövecses, 2010; Lakoff & Johnson, 2011) metaphors have gained ground in linguistic research. They offer insight into the representation of thoughts and are therefore of interest not only for linguists, but psychologists and neuroscientists as well. Metaphor is defined as the understanding of one domain in terms of another. By using metaphors, attributes of one concept (*the source*) are transferred to another one (*the target*), enriching the target’s semantic field with connotations of the source. In most cases, the source domain is more concrete and therefore enhances the understanding of a more abstract target domain. As an example, the taste word “bitter” describes an otherwise hard to verbalize emotion. As emotions are strongly sensory based and placed somewhere between concrete and abstract concepts (Connell, Lynott, & Banks, 2018; Vigliocco et al., 2014), they give rise to various metaphorical expressions (Gibbs, 2002; Kövecses, 2010; Kövecses & Benczes, 2010), many of which belong to the source domain of the human senses (Kövecses, 2019).

The relationship between emotion and language is still a topic of debate in which recent theories assign language a special role in the development and differentiation of emotion concepts in the human brain. According to the theory of constructed emotions, emotions are not hard-wired into the brain, but emotion concepts develop over time, and are formed by cultural and social experiences as well as language (Barrett, 2006, 2017; Lindquist, 2017). According to this theory, humans experience affect, i.e. the intensity and (un-)pleasantness of an incoming stimulus, universally, whereas the resulting emotion concepts depend on both personal experiences and cultural and linguistic background. Neuroimaging results support the important role of language in emotion processing. In an fMRI meta-analysis, Brooks and colleagues (Brooks et al., 2017) examined activation patterns for unspecific affect words and specific emotion terms. Heightened amygdala activation showed experience of affect whereas the semantic network was more active when emotion terms supported the conceptualization of specific emotions. Emotion concepts seem to become more distinct the more mental words a person uses to describe these emotions (Barrett, 2017). Such emotion terms, like “anger” or “sadness”, activate specific emotion categories and help in classifying the affect someone is experiencing. Research shows that emotion words enhance emotion perception (e.g., Gendron, Lindquist, Barsalou, & Barrett, 2012; Lindquist & Gendron, 2013) and that naming and differentiating emotions is associated with mental well-being (Demiralp et al., 2012; Lennarz, Lichtwarck-Aschoff, Timmerman, & Granic, 2018). It is often the case, especially in emotional situations, that people use figurative language instead of literal emotion terms (e.g., Fainsilber & Ortony, 1987; Lee, 2018), which further enriches emotion processing and experience. For example, figurative language was found to be used more often to express intense emotional states than mild ones (Fussell & Moss, 1989). In these situations, figurative language might be chosen because metaphors seem to be more emotional than literal expressions (Citron & Goldberg, 2014; Gibbs, 2002; Mohammad, Shutova, & Turney, 2016). Additionally, sometimes only figurative language can express certain feelings and make them more accessible, like metaphors of darkness and weight that are often used when people suffering from depression talk about their illness (Charteris-Black, 2012).

### Internal state language

Since metaphors are used frequently when referring to abstract concepts or talking about emotional states, internal states present rich target domains for metaphors. Humans verbalize internal processes with internal state language (ISL), which is an important premise to communicate how they are feeling or what they are thinking (see Klann-Delius, 2015; Schwarz-Friesel, 2015). ISL is made up of several categories. To convey emotions, language uses various prosodic, morphological, syntactic, and lexical means, including specific emotion terms like “happy” or “angry” (Kauschke, 2019). Furthermore, there are many affective words which carry emotional connotations but do not explicitly denote specific emotions, such as “holidays” or “funeral”. Some researchers claim that those emotional connotations are fully incorporated into the semantic structure of the word (Taylor, 2010). Apart from emotions, evaluations are a large part of ISL. Evaluations express the speaker’s subjective opinion that is controlled by their affective evaluations of situations or events, like “good” or “bad”. Another category that is not typically listed under ISL but that we find important to mention is general atmosphere. General atmosphere as a category includes expressions for vague impressions of situations or events (Kövecses, 2019). This vagueness makes it a rich domain for many metaphors, like “sparks” between two people or “high running tensions”. It could be placed somewhere between an emotion and an evaluation. To convey intentions, obligations, or desires, ISL provides terms like “should” or “wish for”. Terms for mental processes, like “think”, “imagine”, or “believe”, all fall into the category of cognition. Additionally, words for physical perceptions like “hunger” or “tired” are another category of internal state terms. On a continuum, cognitive terms are the most abstract of internal state terms since the concepts behind the expressions are not perceivable with the senses but constructs of the human mind. In contrast to this, physiology, defined as bodily sensations, is comparatively concrete. Emotions can be placed in between abstract and concrete. On the one hand, they originate within the individual’s mind, making them abstract. On the other hand, psychophysiological components of emotions, like body reactions or emotional expressions, are perceivable for oneself and sometimes even others (Kauschke, 2019). Connell et al. (2018) found that sense modalities, especially interoception (i.e., sensations from within the body, like heartbeat or hunger), vision, and hearing are strongly intertwined with emotion concepts. The continuum from physiological states over emotions in the middle to abstract internal states is also visible in language acquisition (e.g., Kauschke & Klann-Delius, 1997; Kristen, Sodian, Licata, Thoermer, & Poulin-Dubois, 2012). In general, research focuses on literal ISL, especially emotion terms and how they are processed (e.g., Bahn, Kauschke, Vesker, & Schwarzer, 2018; Citron, 2012; Hofmann, Kuchinke, Tamm, Võ, & Jacobs, 2009; Kousta, Vinson, & Vigliocco, 2009; Kuchinke & Mueller, 2019) . Given that affective language is also highly figurative (Schwarz-Friesel, 2015), and only a few empirical studies have addressed metaphors for internal states so far (e.g., Kauschke, Mueller, Kircher, & Nagels, 2018), the present study aims to provide a stimulus set for empirical research to bridge that gap.

### Metaphors and human senses

In many languages human senses are a common source domain for internal state metaphors. Metaphor scholars (e.g., Kövecses, 2019; Kövecses & Benczes, 2010; Lakoff & Kövecses, 2012) have found a great number of conceptual metaphors, i.e. conceptual mappings, for internal states from the source domain based on the senses. A prominent example is the conceptual metaphor AFFECTION IS WARMTH that is based on the sense of touch/temperature. Many metaphorical expressions (“a warm hug”, “a cold-hearted person”, etc.) arise from this conceptual metaphor and suggest a connection between warmth and affection. As another example of a conceptual metaphor, UNDERSTANDING IS SEEING transfers the properties of vision to an abstract mental process, giving rise to expressions like “I see” as a way to express understanding. Kövecses (2019) made a case for the conceptual metaphor EMOTION IS PERCEPTION, illustrating with various examples how the senses are fruitful sources for verbalizing emotions. Additionally, he proposes that the senses might be the only source domains to metaphorically conceptualize the general atmosphere of something. For instance, *smell* and *sound* provide examples like “it had the smell of treason” or “she improved the tone of the meeting”. An explanation for this may be found in embodiment theories that assume all conceptual knowledge, including language, is grounded in the body and how the body navigates the world (e.g., Barsalou, 1999; Kiefer & Pulvermüller, 2012; Pulvermüller, 2005). Sensorimotor information is an important factor in the processing of language. Words that are more strongly perceptually based can be recognized as words more quickly (e.g., Lynott, Connell, Brysbaert, Brand, & Carney, 2020; Siakaluk, Pexman, Aguilera, Owen, & Sears, 2008), and perceptual strength seems to be an additional predictor for variance in lexical (e.g.,Connell & Lynott, 2012; Juhasz & Yap, 2013) and semantic (Pexman, Muraki, Sidhu, Siakaluk, & Yap, 2019) processing. Using transcranial magnetic stimulation (TMS) and functional magnetic resonance imaging (fMRI), studies on the processing of action and body part metaphors (Boulenger, Hauk, & Pulvermüller, 2009; Chen, Widick, & Chatterjee, 2008; Desai, Binder, Conant, Mano, & Seidenberg, 2011; Desai, Conant, Binder, Park, & Seidenberg, 2013; Lacey et al., 2017; Reilly, Howerton, & Desai, 2019) and on taste, texture, and smell metaphors (Citron & Goldberg, 2014; Lacey, Stilla, & Sathian, 2012; Pomp et al., 2018) yielded further results supporting conceptual grounding. Comparing literal (e.g., “bad day”) to metaphorical (e.g., “rough day”) language, activation patterns revealed neural activity in the respective (motor, gustatory, sensory, olfactory) brain areas.

### Control factors for research on metaphor processing

As previously shown, empirical studies on metaphorical processing have used a variety of behavioural and/or neuroscientific methods. All of these methods produce the need for well-controlled verbal stimuli. To account for this need, linguistic databases were created that present values for psycholinguistic variables, such as word frequency, familiarity, or age of acquisition, mostly based on normative ratings. Especially within internal state language, affective variables play an additional important role. Relevant affective variables that belong to emotion language are emotional valence, that is, how pleasant or unpleasant or how positive or negative an event or stimulus is, and arousal, which indicates how intense an event or stimulus is (e.g., Barrett, 2006; Bradley & Lang, 1999; Russell, 1980). Both valence and arousal have been found to influence word-processing on behavioural (e.g., Kever, Grynberg, Szmalec, Smalle, & Vermeulen, 2019; Kousta et al., 2009) and neural levels (e.g., Citron, Weekes, & Ferstl, 2013; Kuperman, Estes, Brysbaert, & Warriner, 2014; Pauligk, Kotz, & Kanske, 2019). However, databases that include psycholinguistic and affective variables for metaphors are rare. For example, the Berlin Affective Word List (BAWL, Võ et al., 2009) contains psycholinguistic and affective variables for affective words, but no metaphors. On the other hand, metaphor databases (e.g., Bambini, Resta, & Grimaldi, 2014; Cardillo, Schmidt, Kranjec, & Chatterjee, 2010; Cardillo, Watson, & Chatterjee, 2017; Katz, Paivio, Marschark, & Clark, 1988) lack affective variables and/or the metaphors’ literal meanings. So far, research on metaphor processing has either ignored affective variables, or needed to conduct pre-study questionnaires, losing time and resources. Recently, Citron and colleagues (Citron, Lee, & Michaelis, 2020) made available a database for German conceptual metaphors, which provides both psycholinguistic and affective values important for empirical metaphor research. This database (called COMETA) consists of 60 metaphorical expressions covering various source and target domains as well as their literal equivalents, and is the first to present a broad overview of a wide range of conceptual metaphors for the German language. However, the COMETA database avoids mention of emotional states or their metaphorical renderings, whereas this target domain is at the centre of the database presented in the present paper.

To further support empirical research on the verbalization of internal states, we built a metaphor database with a focus on internal states as the target domain. For now, we used German metaphors originating from the source domain “human senses” to provide stimuli that can be used in both embodiment and figurative language research. Our database therefore covers the conceptual metaphor INTERNAL STATES ARE SENSATIONS. Sensations are the first part of our database for internal states, with additional source domains being currently rated. The database of metaphors for internal state terms (MIST) provides norms for the agreement between (mostly) one-word metaphors and their literal counterparts, i.e. their synonymy, as well as norms for emotional valence, arousal, and familiarity. The database as well as supplementary materials can be found at: https://osf.io/gtk52/. Using the database’s stimuli, we then conducted a sentence completion experiment in which participants had to choose a literal or figurative ending for a sentence. In the following, we outline the construction of the database and ratings, as well as the results and application within the sentence completion experiment.

## Construction of the database “Metaphors for Internal State Terms” (MIST)

### Method

#### Participants

In total, 643 German native speakers of various dialectal origin, gender, ages, and educational backgrounds (see Table 1) rated the four variables agreement, valence, arousal, and familiarity in eight separate online questionnaires, resulting in a minimum of 80 participants per questionnaire (see Table A in the supplementary material for a breakdown of participant data for each questionnaire). The division into two questionnaires per variable was necessary to keep the rating workload within a reasonable timeframe for the participants. Despite the unequal distribution, there were no significant differences between the sexes, age, or education groups when comparing mean ratings. Participants were recruited on social media channels and through university-wide appeals via emails and leaflets. A raffle for cinema vouchers and other prizes encouraged motivation for participation.

**Table 1:** Total number of participants listed by gender, age, and education equivalent to the German school system

Gender		Age		Highest education	
Female	489	< 20	42	No degree	3
Male	148	20–25	221	Secondary Education	29
Other	6	26–30	191	A-levels	243
		31–35	49	University degree	368
		36–40	40		
		41–50	44		
		51–60	44		
		> 60	12		

### Materials

The stimulus set includes 168 metaphorical expressions of the target domain ‘internal states’ as described in the introduction. For this project, we divided internal states into the five subcategories *emotion*, *cognition*, *evaluation*, *general atmosphere,* and *physical states*. An expert team of three trained linguists sorted all metaphors into the five target domains. Disagreements were settled by discussion and consent. This database focuses on the senses as source domains for metaphorical expressions. In addition to the five standard senses, we added the categories of colour and temperature because of the high number of examples falling into these categories. Therefore, the seven source domains in this database are *vision, colour, hearing, smell, taste, touch*, and *temperature*.

Complementary to the metaphorical expressions, the database includes literal counterparts. Both metaphors and their equivalents were collected using two approaches: First, German dictionaries (Duden online 2013; DWDS 2020) were scoured for entries regarding the source domain human senses and metaphorical meanings were extracted, if there were any. As the second approach, we focused on concepts of the target domains. Metaphorical synonyms for internal state terms were taken from synonym dictionaries (Duden online; Woxikon, 2020). Three trained linguists with German as their native language selected the final metaphors and two possibilities for literal counterparts that they thought best fit.

As a last step, the stimuli were included in a two-sentence context that differed only in the metaphoricity of the last part of the sentence to ensure a metaphorical understanding of the target words. The introductory sentence provided the context, whereas the second sentence included the metaphor or literal equivalent. Wherever possible, both metaphors and literal equivalents consisted of only one word. Both sentences together were between 12 and 18 syllables long. Examples of internal state metaphors in context using the senses as source domains can be seen in Table 2.

**Table 2:** Examples of internal state metaphors and their literal counterparts in context sentences

Source domain	Metaphorical expression	Target domain	Literal expression	Context
Vision	hell(e) *bright*^1^	Cognition	intelligent *intelligent*	Er wusste nicht viel. Daher galt er nicht als hell(e) / intelligent. *He didn’t know very much. Therefore, he wasn’t considered very bright / intelligent.*
Colour	blau *blue*	Physicals	betrunken *drunk*	Sie tranken zwei Flaschen Wein. Jetzt sind sie blau / betrunken *They drank two bottles of wine. Now they are blue / drunk.*
Smell	stinkig *stinky*	Emotion	wütend *angry*	Den Vorwurf ließ er sich nicht machen. Er wurde stinkig / wütend. *He opposed the accusation. He got stinky / angry.*
Taste	bitter *bitter*	Emotion	ärgerlich *annoying*	Sie haben das Spiel knapp verloren. Das ist bitter / ärgerlich *They lost the game by a short margin. That’s bitter / annoying.*
Touch	schmierig *greasy*	Evaluation	unangenehm unpleasant	Er soll sie in Ruhe lassen. Er ist schmierig / unangenehm. *He needs to leave her alone. He is greasy / unpleasant.*

^1^English translations of the original German stimuli are provided in italics

### Procedure

Stimuli were presented in two phases via online questionnaires realized by SoSci Survey (Leiner, 2019). The first round of ratings assessed the agreement between the metaphorical expression and two possible literal counterparts, e.g. “intelligent (*intelligent*)” and “klug (*smart*)”, as possible counterparts for the metaphor “hell(e) (*bright*)”). Subjects were instructed to rate both literal expressions in accordance with the metaphorical expression on a Likert scale from 1 (*rather incongruous*) to 7 (*high agreement*). A metaphorical expression and its possible literal counterpart were considered an item pair. An item pair with an agreement rating of 7 would therefore be highly synonymous. The stimuli were presented in pseudo-randomized order. At two points within the survey, an attention question was added. Failure to correctly answer these questions resulted in the exclusion of the participant’s dataset. All in all, the rating of one questionnaire took around 20 minutes. A minimum of 80 valid datasets per survey was needed to close participation. After the first round, the literal expression with the higher mean was chosen as the official literal counterpart for the metaphor, and moved on to the second phase of ratings.

**Fig. 1:**
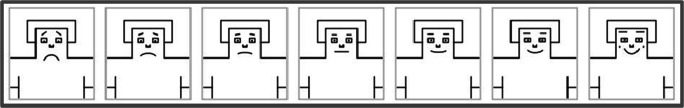
Self-Assessment-Manikin Scale "valence" (Bradley & Lang, 1994) modified to 7-point scale (Irtel, 2007)

The second phase of ratings included different participants and began only after all ratings and calculations of phase 1 were finished. It consisted of the assessment of the psycholinguistic variable of familiarity and the affective variables of emotional valence and arousal for both the metaphorical expressions and their literal equivalents. Because metaphorical meanings cannot be extracted from word frequency databases, we decided to rate familiarity as a variable. Research shows a strong correlation between word frequency and familiarity (Rapp, 2013; Tanaka-Ishii & Terada, 2011). For the purpose of rating, each sentence was presented twice on different pages of the questionnaire; once with the metaphorical ending and once with the literal ending that was selected as the official counterpart after phase 1. Participants were instructed to rate all variables as intuitively as possible. During no part of the survey did participants hear or read the word “metaphor” or “figurative language”, to avoid priming their judgements of the different expressions. All variables were presented in pseudo-randomized order and rated on 7-point scales. Emotional valence, i.e. positivity/negativity of the stimulus, was rated from −3 (very negative) to +3 (very positive). Arousal, i.e. intensity, was rated from 1 (very calm) to 7 (highly arousing). In addition, the Self-Assessment Manikin graphics (SAM, Bradley & Lang, 1994; modified version Irtel, 2007), which depict the continuums of emotional valence and arousal in a comic figurine, were presented at the top of each page. This was done to facilitate understanding of the variables. Examples of the valence graphics can be seen in Fig. 1. Familiarity was rated from 1 (not familiar) to 7 (highly familiar) and described as how often the participants were in contact with the critical expression, either in written or spoken language. Again, two attention questions were added in each questionnaire, and ratings were closed after a minimum of 80 valid datasets. At the beginning of each survey, the respective variable was explained, and examples based on sentences that were not part of the stimuli set were given. The complete instructions and graphics can be found in the supplementary materials. In all, each expression was rated by the minimum of 80 native speakers on agreement, emotional valence, arousal, and familiarity, resulting in a total of 643 valid individual participants (see Table A, supplementary material). A further 193 participants had to be excluded for failing to answer the attention question correctly.

### Results

All calculations were done using IBM SPSS version 26 with Bonferroni corrections applied manually when necessary. For each item, mean values, minimum and maximum scores, and standard deviations were calculated for the variables *agreement*, *valence*, *arousal*, and *familiarity*. The set shows a broad distribution of high and low intensity as well as positive and negative items. Variable values for each expression can be seen in the database and used accordingly for empirical research.

**Fig. 2:**
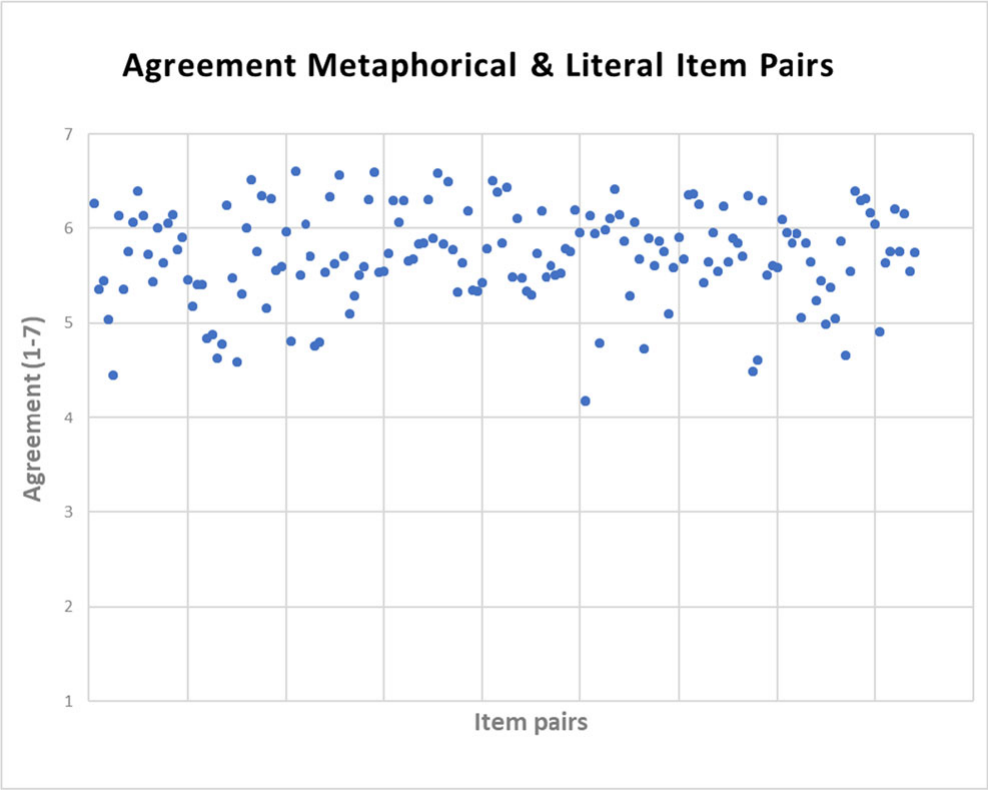
Mean agreement values between metaphorical and literal expressions on a scale from 1 to 7. Each dot on the x-axis represents one of the 168 item pairs

Agreement ratings indicated high similarity in meaning between the metaphorical and literal expressions. On the 7-point scale, where 7 indicates the highest similarity, the item pairs (i.e., the sentences with their metaphorical ending and their respective literal counterpart) ranged between 4.18 and −6.6, with a mean agreement of 5.71 (*SD* 0.5; see Fig. 2 for all 168 item pairs).

To further test for emotional and familiarity differences between the expressions, we calculated mean values and standard deviations of the variables *valence*, *arousal*, and *familiarity* for both groups (see Table 3). Since the expressions received high similarity ratings, we expected only small differences between the groups.

**Table 3:** Mean values and standard deviations for agreement, valence, arousal, and familiarity for metaphors and literal expressions. Scales ranged from −3 to +3 for valence and 1 to 7 for arousal and familiarity

Variable	Metaphorical expressions			Literal expressions		
	*M*	*SD*	*Range*	*M*	*SD*	*Range*
Valence	−0.16	1.34	−2.27 - 2.48	−0.09	1.30	−2.36 to 2.49
Arousal	3.78	0.94	1.81 - 6.09	3.70	0.97	1.54–5.83
Familiarity	5.72	0.80	2.59 - 6.94	6.44	0.53	3.59–6.98

**Fig. 3:**
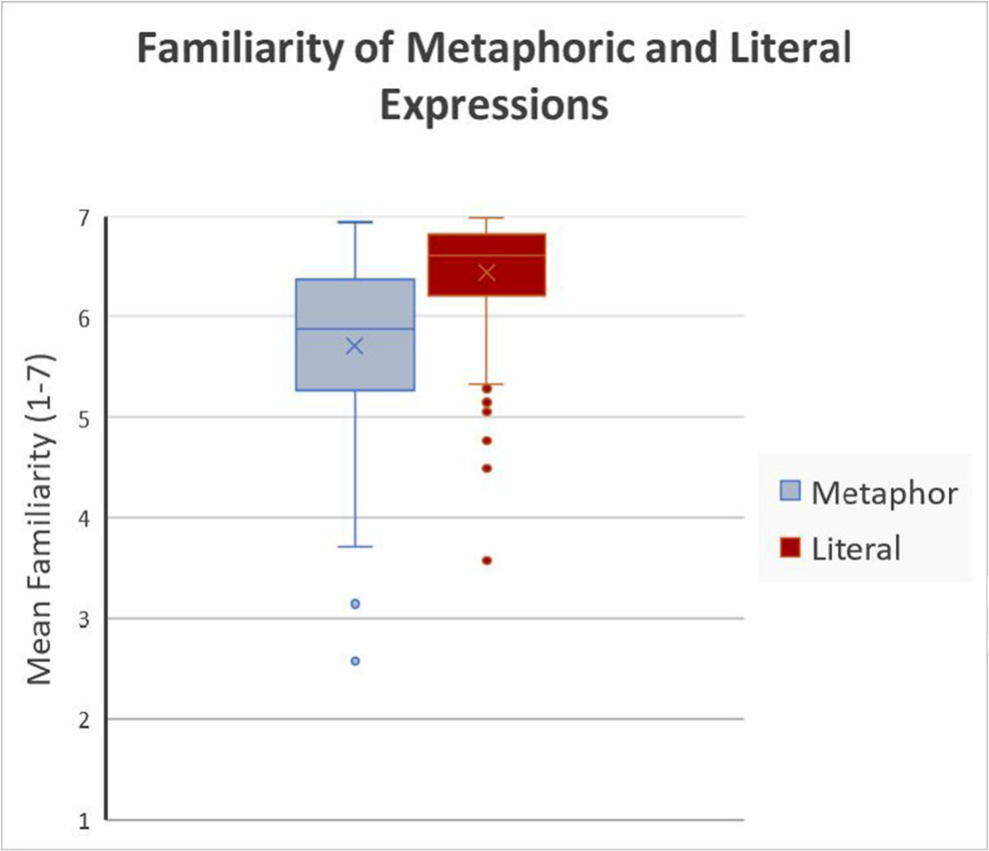
Familiarity of metaphorical and literal expressions on a scale from 1 to 7

Except for arousal, none of the variables was normally distributed. Valence had a natural bimodal distribution whereas familiarity was strongly negatively skewed, since all items were rather frequently used German expressions (see supplementary material for distribution figures). To avoid alteration of the natural distribution of the data, we refrained from any transformation. With 168 items, the sample size is large enough that violation of the normality assumption can be ignored when calculating *t* tests (Boneau, 1960; Salkind, 2010). Results showed that metaphors were significantly less familiar than literal expressions, *t*(334) = −9748, *p* < .001 (Fig. 3). Neither valence nor arousal differed significantly between the types of expressions. To further examine the lack of a statistical difference in the variables valence and arousal, we used a two one-sided tests (TOST) procedure that tests for equivalence and rejects the presence of a smallest effect size of interest (SESOI, Lakens, Scheel, & Isager, 2018). As there is, so far, only one study we could have used as a reference for an effect size (Citron et al., 2020), we chose to follow Brysbaert (2019), who found an effect size of *d* = .4 as the most reasonable estimate for an effect size when there is scarce evidence available. He based his estimate on replication studies and meta-analyses that found *d* = .4 as the average effect size in psychological research (Bosco, Aguinis, Singh, Field, & Pierce, 2015; Camerer et al., 2018; Gignac & Szodorai, 2016; Open Science Collaboration, 2015). It is also noted, however, that many studies report even smaller effect sizes, and a great number of studies with smaller effect sizes remain unpublished due to publication bias. On this basis, we narrowed our equivalence bounds below the average effect size to *d* = .3 with an alpha of 0.5. The TOST procedure for Welch’s *t* test for independent samples, with equivalence bounds of ΔL = −0.3 and ΔU = 0.3, was significant for valence, *t*(334) = 2.29, *p* = .011, and arousal, *t*(334) = −1.96, *p* = .025. Based on the equivalence tests and the non-significant tests of difference, we can conclude that the observed effects, i.e. the mean valence and arousal values for metaphors and literal expressions, are statistically equivalent.

In a next step, we examined the relationship between the variables of the database over all items as well as metaphorical and literal expressions separately. By omitting the negative sign in front of the valence values, we transformed the original valence values into a new variable “emotionality” (e.g., −2.54 became 2.45). Therefore, emotionality displays the extent of the target words’ emotional valence independent of negativity or positivity and can be used as an emotionality marker. Pearson’s correlations were calculated between the variables *valence, emotionality, arousal*, and *familiarity*. Following the literature on affective variables (e.g., Bradley & Lang, 1999; Citron et al., 2020; Citron, Cacciari, et al., 2016a; Võ et al., 2009), we expected a quadratic relationship between valence and arousal values; i.e. extreme valence values, independent of polarity, should correlate with high arousal values. This quadratic relationship was expected to be slightly asymmetric, since negative expressions seem to display higher arousal levels than positive ones. All residuals in the following analyses followed a normal distribution. The results of the correlation analyses over all items, as well as metaphors and literal expressions separately, are presented in Table 4.

**Table 4:** Correlation matrix of Pearson correlations between all variables over all items (ALL) and metaphorical (MET) and literal (LIT) expressions separately

	VALENCE			EMOTIONALITY			AROUSAL			FAMILIARITY		
	All	MET	LIT	ALL	MET	LIT	ALL	MET	LIT	ALL	MET	LIT
VALENCE	1	1	1	.07	.07	.07	− .20**	−.19*	−.21**	.15*	.18*	.17
EMOTIONALITY	.07	.07	.07	1	1	1	.11	.09	.11	.04	.11	.01
AROUSAL	− .20**	−.19*	−.21**	.11	.09	.11	1	1	1	.03	.12	−.04
FAMILIARITY	.15*	.18*	.17	.04	.11	.01	.03	.12	−.04	1	1	1

*p < .05, **p <.01

Familiarity correlated positively with valence—driven by the metaphorical expressions—indicating that positive metaphorical expressions were marginally more familiar than the negative ones. Arousal correlated negatively with valence, showing that negative expressions, both literal and metaphorical, were slightly more arousing than positive ones. Both correlations, however, were rather weak. Surprisingly, no significant correlation between arousal and emotionality was found. Since familiarity correlated with valence but not arousal, we conducted Pearson partial correlations to further examine the relationship between the affective variables. When controlled for familiarity, Pearson partial correlations still revealed a significant negative correlation between arousal and valence for all expressions, and for metaphorical and literal separately (metaphorical: *R* = −.215, *p* = .005, literal: *R* = −.207, *p* = .007, all: *R* = −.206, *p* < .001).

**Fig. 4:**
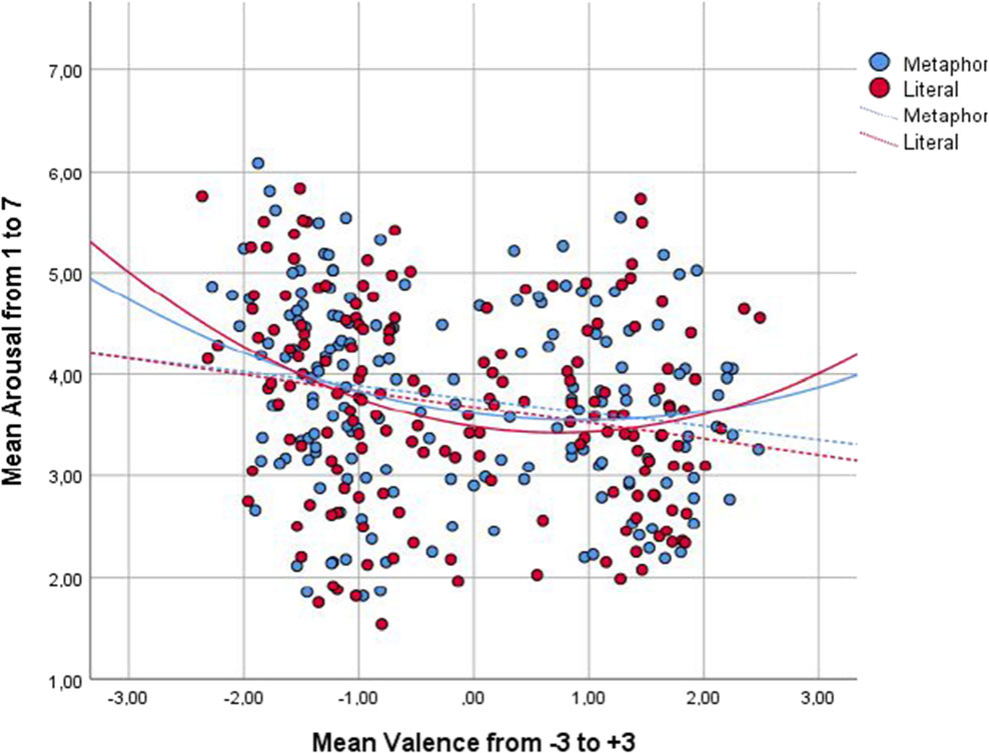
Distribution and relationship of mean valence and arousal values for metaphorical and literal items. For all items, a negative linear and a positive quadratic relationship can be observed

In a next step, we modelled regression analyses to examine how valence could explain variance in arousal. As previous research suggests asymmetric quadratic relationships, we calculated quadratic and linear models, expecting valence of both polarities to have an effect. Since correlations differed between metaphorical and literal expressions, regressions were modelled separately for the two groups as well as over all items. The results showed a significant influence of valence on arousal in both linear and quadratic regression analyses (Fig. 4). Quadratic relations revealed that arousal levels increased the further valence negatively or positively deviated from zero. Furthermore, the negative linear regression illustrated that negative valence produced higher arousal levels than positive valence. In all, valence could only explain up to 5.5% of variance in arousal values. No other variables had a significant influence. Even though no significant influence of the type of item (metaphorical/literal) could be observed, models within the literal items had a better fit than within the metaphors, indicating higher variability within the metaphors. Results of the regression models can be seen in Table 5.

**Table 5:** Results of the regression analysis

Items	Model	Variable	*B*	*SE B*	*β*	*R*^*2*^ *adjusted*
Metaphorical	Linear	Constant	3.756	0.07		.030*
		Valence	−0.13	0.05	−.190	
Literal	Linear	Constant	3.68	0.07		.044**
		Valence	−0.16	0.06	−.210	
All items	Linear	Constant	3.72	0.05		.037***
		Valence	−0.15	0.04	−.201	
Metaphorical	Quadratic	Constant	3.62	0.12		.036*
		Valence	−0.14	0.05	−.204	
Literal	Quadratic	Constant	3.49	0.12		.055**
		Valence	−0.17	0.06	−.222	
All items	Quadratic	Constant	3.55	0.09		.051***
		Valence	−0.16	0.04	−.215	

*p < .05, **p <.01, and ***p < .001

As a final step, internal consistency between the participants (*n* ≥ 80) was calculated using Cronbach’s alpha, with ratings for *agreement*, *valence*, *arousal*, and *familiarity* as variables. Results of the interrater reliability are shown in Table 6. Since each variable was assessed in two separate questionnaires, one alpha value was computed for each set. In all, there was a very high internal consistency, demonstrating high consistency within the ratings across participants.

**Table 6:** Cronbach’s alpha values for each questionnaire set

Variable	*N*	Cronbach’s alpha
Agreement 1	80	.980
Agreement 2	80	.973
Valence 1	80	.886
Valence 2	80	.964
Arousal 1	80	.986
Arousal 2	81	.972
Familiarity 1	80	.984
Familiarity 2	82	.980

## Sentence completion experiment

Using part of the database’s stimuli in a sentence completion experiment, we tested subjects’ individual preference for literal or metaphorical expressions. We expected a preference for literal language since literal expressions were more familiar and common in everyday language. Participants were instructed to listen to the context sentences followed by the literal and metaphorical ending. Via button press, they indicated which ending they would choose to complete the sentence. Following the experiment, participants were tested on their metaphor processing abilities.

### Method

#### Participants

Twenty-three German native speakers were recruited via a university-wide call for participation. Two had to be excluded due to medication and technical malfunctions, and one scored below the cut-off value in a metaphor comprehension test. Twenty participants, 15 of whom were female, aged 18–62 (*M* = 32.3, *SD* = 13.83), were included in the analysis. None were diagnosed with neurological, psychological, hearing or language problems. Subjects were paid €5 for their participation.

### Materials

Stimuli were taken from the MIST database. In all, 100 expressions divided into the five categories sight, hearing, smell, taste, and touch were used and statistically controlled for mean differences in emotional valence, arousal, and familiarity. The results showed no significant differences for valence and arousal between metaphorical and literal expressions. Familiarity, however, differed significantly between the two, *t*(198) = −7778, *p* < .001 (see Table 7). The stimuli were then recorded by a linguistically trained speaker using Audacity version 2.3.0. Metaphorical and literal endings were articulated after a short pause following the introductory context. This way we avoided confounding articulatory artefacts. The speaker was instructed to produce metaphorical and literal endings in an unchanged voice with steady loudness and pitch. Recordings were cut into three audio files per sentence: the carrier sentence, and the metaphorical and literal expressions. Subsequent phonetic analyses comparing mean pitch (in Hz) and intensity (in dB) values across metaphorical and literal endings showed no significant differences between the two categories (see Table 7).

**Table 7:** Mean values and standard deviations of valence, arousal, and familiarity and the phonetic parameters pitch and intensity for the stimuli used in the sentence completion experiment

Variable	Metaphorical expressions		Literal expressions		*p*
	*M*	*SD*	*M*	*SD*	
Valence	−0.16	1.34	−0.09	1.30	n.s.
Arousal	3.78	0.94	3.70	0.97	n.s.
Familiarity	5.72	0.80	6.44	0.53	.001***
Pitch	194.71	21.09	195.03	22.89	n.s.
Intensity	70.11	2.41	70.26	2.53	n.s.

### Procedure

Participants were seated comfortably in front of a 22-inch monitor wearing headphones. The experiment was implemented and presented using OpenSesame version 3.2.7. Participants were instructed both verbally and on screen to choose their preferred ending of a sentence. After a short trial phase, the experiment began. Items were presented auditorily in five blocks with 20 sentences each. Sentences and metaphorical and literal endings were presented in pseudo-randomized order, with two conditions: the sense categories should be similarly distributed across the five blocks, and literal and metaphorical endings should not be presented in the same order more than four times. In all, the experiment lasted between 15 and 20 minutes. After the experimental part, participants completed subtest 3 of the German version of the Protocol Montréal d’Evaluation de la Communication (MEC, Ska et al., 2016). The MEC is a diagnostic tool used to evaluate pragmatic communication disorders. Subtest 3, Comprehension of Metaphors, tests metaphor processing accuracy. Participants listen to 10 known and 10 novel metaphors and are asked to explain their meaning. A two-point system is used to evaluate the accuracy of the explanation, resulting in a maximum of 40 points. If the participant fails to answer correctly, three options are presented, of which they can choose one. The dataset of one subject who scored below the cut-off value (<34 points) was excluded from further data processing.

### Results

**Fig. 5:**
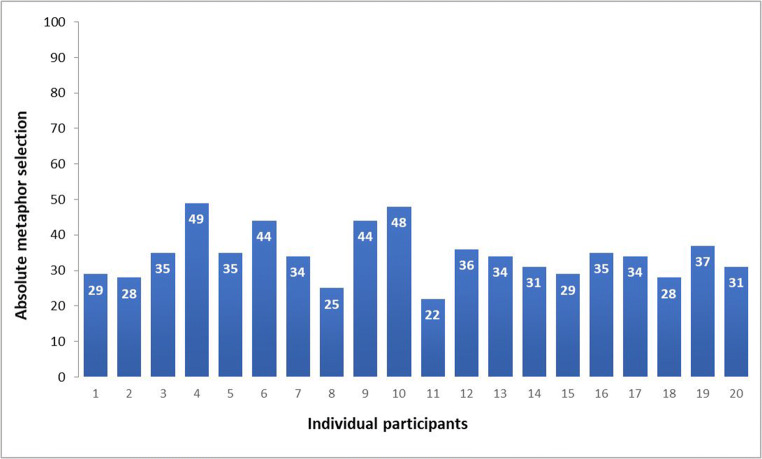
Absolute metaphor selection (of 100 possible) in the sentence completion experiment

In an item-based analysis, absolute and mean percentage of metaphor selection were calculated for each item. Additionally, we calculated absolute and mean percentage of metaphor selection over all items. Participants showed a preference for literal expressions, as they chose 36.6% of the metaphorical and 63.4% of the literal sentence endings (*M* = 36.60 (metaphorical), *M* = 63.4 (literal), SD = 26.18). Gender revealed no significant effect. In a subject-based analysis, high variation could be observed (Fig. 5). The results of the MEC were high overall, with a mean of 38.2 points out of 40 (*SD* = 1.79). Testing the hypothesis that high MEC scores would correlate positively with metaphor selection, we calculated one-tailed Pearson’s correlations. Results showed a positive correlation of metaphor selection and the results of the MEC metaphor test, *r* = .426, *p* = .030. Higher scores in the MEC correlate with a higher absolute selection of metaphorical endings.

To further analyse the influence of familiarity on the observed preference for literal endings, we conducted item-based analyses. All residuals in the following analyses were normally distributed. Pearson partial correlations were calculated between absolute metaphor selection and familiarity, with *metaphoricity* as a control variable. Results showed a significant positive correlation for *selection* × *familiarity* (*R* = .595, *p* < .001) within both literal and metaphorical expressions, indicating a higher number of selections the more familiar an expression was. Since familiarity differed significantly between the two groups, we used the absolute metaphor selection in a univariate analysis of covariance (ANCOVA) with *metaphoricity* as one factor (including the two conditions *literal* and *metaphorical*) and familiarity ratings as the covariate. The results showed highly significant effects for the covariate familiarity, *F*(1) = 58.84, *p* < .001, as well as the factor *metaphoricity*, *F*(1) = 12.188, *p* < .001. Taking familiarity into account, our results demonstrate the significant preference for literal language within this sentence completion experiment.

## General discussion

The aim of this study was to provide a dataset, called the MIST database, which includes ratings for internal state metaphors to be used in various branches of research. For this purpose, we compiled a database including 168 metaphorical items from seven sense categories serving as source domains. The results are statistically controlled German metaphorical and literal expressions that differ only in their familiarity. The higher familiarity of the literal expressions corresponds to findings from different languages, showing that literal expressions are more frequently read, heard, or spoken than metaphorical expressions (e.g., Cardillo et al., 2010; Cardillo et al., 2017; Citron et al., 2020). Even though on the neural level, metaphors were found to be more emotional (Citron & Goldberg, 2014; Citron, Güsten, Michaelis, & Goldberg, 2016b), there were no significant differences in our subjective ratings of arousal or valence values. Citron et al. (2020) obtained similar results for ratings of metaphorical and literal sentences. In contrast, metaphorical stimuli in stories (Citron et al., 2020) registered higher arousal values for figurative than literal expressions. This points to differences in emotionality between metaphorical sentences and stories that could derive from the context in which figurative language appears. Another explanation for the heterogeneous findings might be that Citron and colleagues avoided emotion terms as literal equivalents, whereas we actively compared metaphorical and literal expressions for emotional and other internal states. It might be the case that literal emotion terms already carry high emotion values that do not increase by using metaphors.

Correlation analyses showed significant negative correlations between valence and arousal values over all stimuli, as well as within metaphorical and literal expressions separately. No significant correlations were found for arousal with emotionality, independent of polarity. Even though high arousal values for high emotionality is a result that has been reported previously (Bradley & Lang, 1999; Citron et al., 2020; Citron, Cacciari, et al., 2016a; Võ et al., 2009), our database does not support this finding, demonstrating that effects for arousal and negative valence exceed effects for arousal and emotionality in general. Including the variables in regression models to examine the dependence of arousal on valence, we observed a significant influence of valence but not familiarity or metaphoricity. Quadratic and linear relationships were significant but overall not very high, further suggesting the independence of arousal values, i.e. arousal cannot be explained by valence alone. This independence strengthens the need to control valence and arousal separately when conducting experimental metaphor research. In addition, it underlines the need for more fine-grained analyses of the relationship between the affective variables.

Agreement between the metaphors and their literal equivalents was high (*M* = 5.71 on a 7-point scale), indicating well-chosen literal counterparts for the metaphorical expressions in our contexts. It should, however, be pointed out that metaphors are not synonyms for literal expressions (Gibbs & Colston, 2012; Gibbs Jr. & Gerrig, 1989). The same metaphor can be used in various contexts with slightly different meanings and connotations; for example, a sweet child, a sweet kiss, a sweet temptation. A comparison with items from the metaphor database COMETA (Citron et al., 2020) exemplifies this finding. Overall ten metaphors were identified that were in both COMETA and MIST (“bitter, köstlich, kalt, schmierig, scharf, einschleimen, hart, weich, hell(e), ge-/versalzen”; *bitter, delicious, cold, slimy, sharp, greasing, hard, soft, bright,* and *salted*). Apart from “intelligent” (*intelligent*) as the synonym for “hell(e)” (*bright*), no literal counterpart was the same. For example, the German metaphor “köstlich” (*delicious*) had the literal equivalents “wunderschön” (*beautiful*, COMETA) and “lustig” (*funny,* MIST). Not only do the equivalents differ, but the rating results do as well. The same metaphor in a different context elicits different affective attributes. One possible explanation could be that metaphors cannot be directly translated into literal language since they simply transfer some affect that gains its meaning only within the semantic context it is related to. Affect is transferred from the source domain but varies depending on the context in which the metaphor is used. The consequence of this finding is that metaphors themselves cannot be rated separately, but only embedded in the context in which they appear. We would therefore like to advise researchers against using the metaphor’s psycholinguistic variables in our database independent of their original context.

Using part of the stimuli in our sentence completion experiment, we observed a preference for literal language over metaphorical language. Even though familiarity correlated positively with selection, ANCOVA results revealed a significant effect for metaphoricity when controlled for familiarity. Literal expressions were selected significantly more frequently than metaphors. There was high variability between the individual participants regarding the proportion of selected metaphors. While they all chose more literal expressions, the proportion of metaphor selection ranged from 22% to 49%, demonstrating distinct individual preferences. Interestingly, higher percentages of metaphor selection correlated positively with the results in the metaphor test, demonstrating a link between the ability to explain metaphorical meanings and a preference for metaphorical language.

The stimuli in this database reflect the conceptual metaphor INTERNAL STATES ARE PERCEPTIONS and are therefore grounded in the sensory domains. Several studies have demonstrated that the processing of sensory metaphors activates respective brain areas (see Citron & Goldberg, 2014; Lacey et al., 2012; Pomp et al., 2018). Proceeding from these results, our database can be used to replicate neurolinguistic findings for taste and smell and also to examine the conceptual grounding of metaphors in vision, hearing, colour, and temperature as well.

Since metaphors are an important part of human language and cognition, future empirical research into the processing of internal state metaphors is of great interest to broaden our understanding of the human brain and mind. Affective disorders could provide additional insights into the relationship between emotion and metaphor processing. Therefore, metaphors provide important links in both neurolinguistics and psychological research to further examine the relationship between language, cognition, and emotion.
